# Identification and Selection of Prospective Probiotics for Enhancing Gastrointestinal Digestion: Application in Pharmaceutical Preparations and Dietary Supplements

**DOI:** 10.3390/nu15061306

**Published:** 2023-03-07

**Authors:** Claudia Cappello, Ali Zein Alabiden Tlais, Marta Acin-Albiac, Wilson José Fernandes Lemos Junior, Daniela Pinto, Pasquale Filannino, Fabio Rinaldi, Marco Gobbetti, Raffaella Di Cagno

**Affiliations:** 1 Faculty of Science and Technology, Free University of Bolzano, 39100 Bolzano, Italy; 2 Human Microbiome Advanced Project, Research & Development, 20129 Milano, Italy; 3 Department of Soil, Plant and Food Science, University of Bari Aldo Moro, 70121 Bari, Italy

**Keywords:** lactic acid bacteria, digestibility, food functionality, anti-nutritional compounds, scoring approach

## Abstract

Our study investigated the effectiveness of 446 strains of lactic acid bacteria (LAB) belonging to different species and isolated from diverse sources (food, human, and animal) as potential probiotic candidates, with the perspective of producing dietary supplements or pharmacological formulations suitable for enhancing gastrointestinal digestion. The survival capability of all the isolates under harsh gastrointestinal tract conditions was evaluated, in which only 44 strains, named high-resistant, were selected for further food digestibility investigations. All 44 strains hydrolyzed raffinose and exhibited amino and iminopeptidase activities but at various extents, confirming species- and strain-specificity. After partial in vitro digestion mimicking oral and gastric digestive phases, food matrices were incubated with single strains for 24 h. Fermented partially digested matrices provided additional functional properties for some investigated strains by releasing peptides and increasing the release of highly bio-accessible free phenolic compounds. A scoring procedure was proposed as an effective tool to reduce data complexity and quantitively characterize the probiotic potential of each LAB strain, which could be more useful in the selection procedure of powerful probiotics.

## 1. Introduction

The occurrence of disorders in the gastrointestinal tract (GIT), such as diarrhea, constipation, ulcerative colitis, irritable bowel syndrome (IBS), and related allergic reactions (e.g., allergic rhinitis), has been highly associated with abnormalities in the microbial abundance in stool samples of diseased people [1]. Currently, antibiotics and synthetic drugs are used to treat these disorders as well as other wide ranges of lethal bacterial infections. Antibiotic therapy is associated with several negative consequences: causing serious adverse effects, failing to discriminate between good and harmful microorganisms, affecting the normal microbiota, resulting in vitamin deficiency, impairing defense mechanisms in the human body, and increasing multi-drug resistances [2]. Extensive studies have been dedicated to substitute antibiotics where probiotics emerged as a potent, natural, and cheap alternative [3,4]. Probiotics are defined as living, non-pathogenic bacteria that, when consumed appropriately, can improve human health, bringing benefits both to the nutritional and therapeutic sides [5,6]. Generally, probiotics work by increasing the production of enzymes that support food digestion and nutrient absorption, improving the morphology of the intestinal-epithelial cells (IEC), boosting the immune system, enhancing the number of beneficial microorganisms in the intestine, promoting the barrier function of the IEC, and preventing pathogens and toxins from adhering to the IEC, impacting on the gut microbiome with a strain-specific level of effectiveness [7]. Nutrition and diets play a significant role in how the human gut microbiota develops from childhood through old life. The lack of long-term investigations on dietary changes and the identification of gut microbiota over time limits the current understanding of how eating habits shape the gut microbiome [8]. Nevertheless, consumers’ perception toward lactic acid fermented foods is steadily growing favorably, owing to their potential relevance for the gut microbiota as well as their health-promoting properties, with fermented foods and beverages accounting for 30% of human diets, with particular attention to side effects that may derive from dysbiotic conditions [9]. High digestibility is a distinctive trait of fermented foods compared to unprocessed foods [10]. Indeed, lactic acid bacteria (LAB) are a diverse group of microbial species involved in food fermentation that have revealed an optimal portfolio of enzymes useful for improving food digestibility [11]. The metabolic and functional labyrinth followed by LAB during foods fermentation may enhance the nutritional value of food (e.g., by increasing the bioavailability of amino acids and releasing bioactive peptides and phenolics derivatives) [11,12] and may reduce anti-nutritional factors (ANFs) such as raffinose [13,14]. These properties assume some of the digestive functions of probiotics and make fermented food a promising source of LAB as prospective probiotics, alongside other ecological niches, provided that the isolates also exhibit high persisting ability in the human GIT [15]. Therefore, we proposed to investigate the survival capacity of 446 strains of LAB isolated from various sources under simulated gastrointestinal conditions. The selection of potential probiotics with distinct metabolic traits may help to design dietary supplements having the capability to improve digestibility as the main functionality. One of the potential approaches for the selection of probiotics may be taking into consideration results from multiple assays. Starting from this, we developed a new strategy to select probiotic LAB able to enhance the digestibility of food components and hydrolyze ANFs through a cumulative score of evaluation that takes into consideration the performance of each LAB strain for all the assays carried out. The study aimed to evaluate the efficiency of potential probiotic candidates and define a multi-species formulation that could cover a wide range of functional features correlated to better food digestibility, which in turn can enhance the host’s overall health. Peptidase activity and raffinose hydrolysis metabolic traits of LAB, as well as their ability to hydrolyze proteins and enhance the bioavailability of phenolic compounds in mimicked digesta of representative food matrices, were assessed in our study.

## 2. Materials and Methods

### 2.1. Bacterial Cultures and Growth Conditions

A total of 446 food-grade LAB strains (with a currently qualified presumption of safety—QPS—status as judged by the EFSA scientific Panels), mainly from lactobacilli, *Pediococcus* spp. and *Leuconostoc* spp., all belonging to the Department of Soil, Plant and Food Science, University of Bari Aldo Moro, Bari, Italy, and the Faculty of Science and Technology, Free University of Bolzano, Bolzano, Italy, were investigated in this study. All strains were previously isolated from animals (*Apis milifera* intestine, *Drosophila melanogaster* intestine), dairy products (cheese, milk), cereals (spelt, oat, and tritordeum), fruits and vegetables (apple, avocado, carrot, cherry, fennel, grape, kiwi, olives, papaya, pineapple, prune, sauerkraut, and tomato), sourdough, and other sources (Table S3). Cultures were maintained as frozen stocks at −20 °C in De-Man-Rogosa-Sharpe (MRS) broth (Oxoid, Basingstoke, Hampshire, UK) medium with 20% glycerol for subsequent analysis. Before their use, the bacteria were propagated twice in MRS brothat 30 °C for 24 h.

### 2.2. Resistance to Simulated Gastric and Intestinal Fluids under In Vitro Conditions

Initially, 446 LAB strains were subjected to simulated gastric and intestinal fluids, as described by Fernández et al. [16], with some modifications. Briefly, 10 mL of stationary-phase-grown cells (24 h) were harvested (7500 rpm, 10 min), washed with physiologic solution (0.9%, NaCl), and resuspended in 5 mL of simulated gastric fluid (SGF), which contains NaCl (125 mML^−1^), KCl (7 mML^−1^), NaHCO_3_ (45 mML^−1^), and pepsin (3 gL^−1^) (Sigma–Aldrich CO., St. Louis, MO, USA) [17]. The effect of the gastric transit on the bacterial culture was controlled by supplementing with 5 mL of reconstituted skimmed milk (RSM) (11% solids, wv^−1^) at an adjusted pH of 3.0. The bacterial suspensions were incubated under anaerobic and stirring (150 rpm) conditions at 37 °C to simulate peristalsis of the gastric transit [17]. Samples for total viable counts were taken after 3 h of incubation. Subsequently, grown bacterial suspensions were collected by centrifugation (7500 rpm, 10 min) and resuspended to the original volume in simulated intestinal fluid (SIF), which contained pancreatin (0.1%, *w/v*) and bile salts (0.15%, *w/v*, Sigma-Aldrich Co.) adjusted to pH 8.0. The suspensions were incubated as above and samples for total viable counts were taken after 3 h of the intestinal transit [18].

### 2.3. Raffinose Hydrolysis

Based on the finding of the first screening, further analysis was carried out only on 44 strains that were highly resistant to GIT conditions. Stationary-phase-grown cells were harvested (7500 rpm, 10 min), washed with physiological solution, and resuspended into Raf-MRS (MRS broth supplemented with 20 g L^−1^ of raffinose) and adjusted to a final cell density corresponding to ca. 7.0 Log CFU mL^−1^. After 24 h of incubation at 30 °C, residual raffinose was measured using the Megazyme kit K-RAFGL raffinose/sucrose/D-glucose assay (Megazyme International Ireland Limited, Bray, Ireland) following the instructions provided by the manufacturer.

### 2.4. Peptidase Activity towards Leu-p-Na and Pro-p-Na Synthetic Substrates

General aminopeptidase type N (EC 3.4.11.11; PepN), and proline iminopeptidase (EC 3.4.11.9; PepI) activities were determined using leucine *p*-nitroanilides (Leu-*p*-Na) and proline *p*-nitroanilides (Pro-*p*-Na) (Sigma) as relatively specific substrates as described by Rizzello et al. [19]. Stationary-phase-grown cells, adjusted to a cell density of ca. 9.0 Log CFU mL^−1^, were centrifuged, washed, and resuspended in phosphate buffer solution (PBS, 50 mM pH 7.0). In 100 µL of cell suspension, the reaction mix (80 μL of 50 mM PBS pH 7.0; 20 μL of 20 mM of Leu-*p*-Na or Pro-*p*-Na substrate in methanol; and 2 μL of NaN_3_, 5%) was added. Samples were incubated under stirring conditions (150 rpm) at 30 °C for 1 h (for Leu-*p*-Na) and 24 h (for Pro-*p*-Na). The reaction was stopped by adding 500 μL of 10% acetic acid. Then, the samples were centrifuged (10,000× *g* rpm, 10 min) and the absorbance (OD_410_) was measured using UV-1800 Spectrophotometer, SHIMADZU. One unit of PepN and PepI activity corresponded to the amount of enzyme required to liberate 1 μmol of *p*-Na min^−1^ under the assay conditions. 

### 2.5. Simulation of Digestion of Targeted Food Matrices

Cheese, bread, tomato, pomegranate, and chickpea flour were chosen as the most representative food matrixes for each food category (animal and plant origin). Partially digested food matrices were prepared as described by Brodkorb et al. [20], with slight modifications. The food matrices were exposed to two sequential digestive phases: the oral and gastric phases. Each food matrix was first mixed with simulated salivary fluid (SSF, 1:1, w w^−1^), CaCl_2_ (1.5 mM), salivary amylase (75 U mL^−1^), and incubated for 2 min while mixing (37 °C, pH 7.0) to simulate the oral phase. The oral bolus was then diluted (1:1, w w^−1^) with SGF, together with CaCl_2_ (0.15 mM), and gastric enzymes (pepsin and gastric lipase, 2000, 60 U mL^−1^) and incubated for 2 h while mixing (37 °C, pH 3.0). The partially digested matrices were then incubated for 24 h at 30 °C with single high-resistant standardized strains (ca. 9 Log CFU mL^−1^) previously selected. Partially digested matrices, without microbial inoculum and incubation, were used as controls. To have an indirect response to the capability of LAB strains to digest the above food matrices, indicators such as total peptides and total free phenolic compounds were evaluated after 24 h of incubation.

### 2.6. Determination of Total Peptides

The peptide concentration in the partially digested food matrices (cheese, bread, and chickpea flour) incubated with the single strains was determined by the o-phthalaldehyde (OPA) method [21], with some modifications. The supernatants were collected by centrifugation (10,000× rpm, 10 min, 4 °C) and analyzed. Briefly, the OPA reaction mix was made by combining the following reagents and diluting to a final volume of 100 mL with distilled water: 50 mL of 100 mM sodium tetraborate; 5 mL of 20% (w w^−1^) sodium dodecyl sulfate; 2.5 mL of OPA solution (50 mg mL^−1^ OPA dissolved in ethanol 96%); 540 μL thiolactic acid. The reaction mixture contained 730 μL of OPA reaction mix and 18.25 μL of peptide sample. Absorbance (OD_340_) was determined. The peptide concentration was calculated from a standard curve prepared using tryptone (0.25 to 1.5 mg mL^−1^) as a reference. Total peptides were expressed as mg of tryptone equivalents per 1 mL of sample. 

### 2.7. Determination of Total Free Phenolic Compounds

The concentration of total free phenolics from the partially digested matrices (tomato, pomegranate, chickpea flour, bread) incubated with single strains was determined according to the official method AOAC 2017.13-2017 [22]. Absorption at a wavelength of 765 nm was measured with a UV-1800 Spectrophotometer (SHIMADZU). The total free phenolic content was expressed as gallic acid equivalents (GAE) per 1.0 L of the sample. 

### 2.8. Scoring Procedure for the Selection of the Most Promising Probiotic Candidates

The evaluation of the best-performing strain candidates was done using a scoring approach. A strain was considered positive (score 1) if the value for a given assay parameter was higher than the third quartile of the total values for such assay; otherwise, it was scored as 0. Then, the proportion of positive scores (%) for a given assay was determined by dividing the total cumulative score for an assay category divided by the number of parameters in the assay: Assay score = (Σ positive score parameter/total number parameter in the assay) × 100.

### 2.9. Statistical Analysis

All analyses were carried out considering triplicates on three biological replicates. Data were submitted for analysis of variance by the general linear model (GLM) of the R statistical package (R, version 1.6.2, available at the Internet address: rcompanion.org/handbook/ accessed on 1 December 2022). A pairwise comparison of treatment means was achieved by a Tukey-adjusted comparison procedure with a *p*-value (*p*) < 0.05 [23].

## 3. Results

### 3.1. Selection of High-Resistant Strains

**Figure 1:**
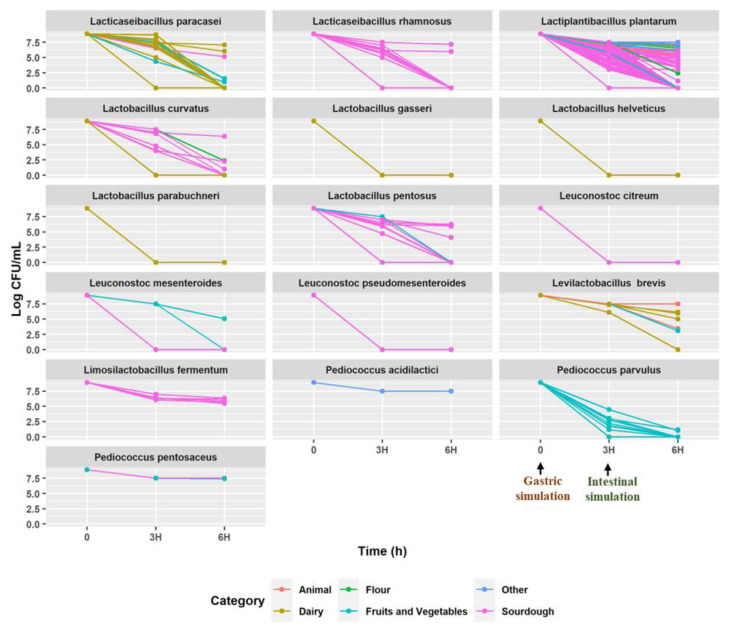
Survival (Log CFU/mL) of 446 lactic acid bacteria under gastric conditions (0 and 3 h) at pH 3 and further intestinal digestion (6 h) at pH 8. The values are the averages of three replicates.

Different species of LAB derived from various sources demonstrated different survival capacities in gastric and intestinal ecosystems (Figure 1). 

The harsh conditions generated by the gastric and intestinal fluid revealed strain-dependent adaptation among species. Strains belonging to the same species of *Lacticaseibacillus paracasei*, *Lacticaseibacillus rhamnosus*, *Lactobacillus pentosus*, *Leuconostoc mesenteroides*, *Levilactobacillus brevis*, *Lactobacillus curvatus*, and *Lactiplantibacillus plantarum* showed inconsistent patterns. While some strains showed no viable counts after gastric simulation (3 h) or after intestinal simulation (6 h), others slightly decreased their cell density throughout the incubation. Almost the same inconsistency was found for *Limosilactobacillus fermentum*, *Pediococcus pentosaceus*, and *Pediococcus acidilactici*, with some strains showing a lower reduction in cell viable count during both gastric and intestinal simulations. All strains of *Lactobacillus parabuchneri*, *Leuconostoc pseudomesenteroides*, *Lactobacillus helveticus*, *Lactobacillus gasseri*, *Leuconostoc citreum*, and *Pediococcus parvulus* were completely inactivated after the first simulation period, showing null values after 3 h of incubation. In detail, only 148 strains were able to adapt to the gastric simulation period (<3 Log cycles reduction), while 62 strains were able to survive the intestinal simulation (Table S1). The viable count of 44 strains was reduced by less than 2 log cycles after 6 h of incubation, demonstrating high resistance to the simulated GIT conditions. These strains belong to *Lev. brevis* (1)*, Lact. paracasei* (1), *L. plantarum* (35)*, Lact. rhamnosus* (1), *P. acidilactici,* (1), and *P. pentosaceus* (5). On the other hand, 18 strains showed intermediate resistance to GIT conditions with a decrease of cell density between 2 and 3 Log cycles after 6 h of incubation. Intermediate resistant strains belonged to the following species: *Lev. brevis* (2), *Lb. curvatus* (1), *Lim. fermentum* (2), *Lact. paracasei* (1)*, Lb. pentosus* (3), *L. plantarum* (8), and *Lact. rhamnosus* (1). For the following screening, the analyses were performed only on the 44 high-resistant strains (Table S2).

### 3.2. Raffinose Hydrolysis

**Figure 2:**
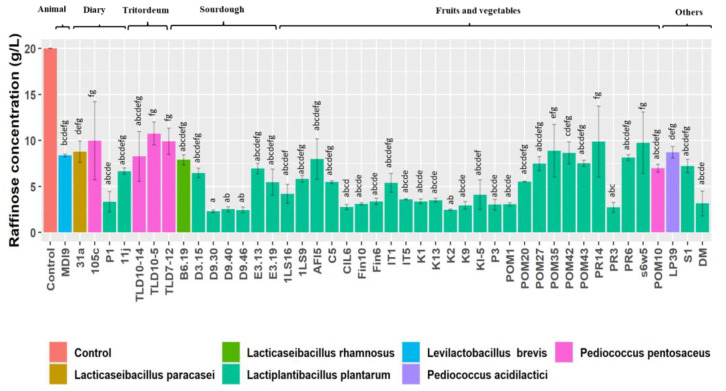
Residual raffinose concentration (g/L) of 44 high-resistant lactic acid bacteria strains, belonging to different species and isolated from different sources, after incubation of Raf-MRS (MRS broth medium supplemented with 20 g/L of raffinose) at 30 °C for 24 h. Data are expressed as the mean of three separate analyses ± standard deviations. Bars with different superscript letters differ significantly (*p* < 0.05).

The 44 high-resistant strains were assessed for their capability to hydrolyze raffinose in an MRS growth medium supplemented with raffinose (Figure 2). 

The inoculum cell density was ca. 7.0 log CFU mL^−1^. After 24 h of incubation, all the strains, mainly *L. plantarum*, showed a considerable (*p* < 0.05) reduction in raffinose content in the growth medium (Figure 2). Indeed, only two strains of *L. plantarum* among the 44 high-resistant strains were found in the first and second quartiles of the raffinose box plot, with high raffinose residuals (Figure S1A). The results showed a variation from a minimum of 2.29 ± 0.13 to a maximum of 10.75 ± 1.25 g L^−1^ of raffinose residuals, measured respectively for *L. plantarum* D9.30 and *P. pentosaceus* TLD10-5 (both isolated from sourdough) (Figure 2).

### 3.3. Peptidase Activity

**Figure 3:**
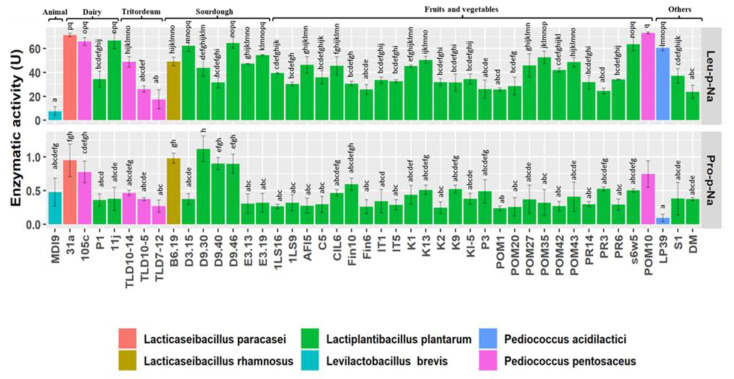
Peptidase activity (U) of 44 high-resistant lactic acid bacteria strains, belonging to different species and isolated from different sources, towards leucine- and proline *p*-nitroanilides. One unit (U) of activity was defined as the amount of enzyme required to liberate 1 μmol of *p*-NA per min under the assay conditions. Data are expressed as the mean of three separate analyses ± standard deviations. Bars with different superscript letters differ significantly (*p* < 0.05).

The potential of the 44 high-resistant strains in catalyzing the hydrolysis of peptide bonds was evaluated through two peptidase activity assays (Figure 3). 

All the strains revealed high general aminopeptidase activity (PepN) on Leu-*p*-Na and to a lesser extent iminopeptidase activity (PepI) on Pro-*p*-Na. The highest aminopeptidase activity was attributed to *P. pentosaceus* POM10 (72.98 ± 0.78 U) and the lowest to *Lev. brevis* MDI9 (7.55 ± 3.63 U). On the other hand, iminopeptidase activity was in the range of 0.09 ± 0.05 to 1.12 ± 0.19 U, respectively, for *P. acidilactici* LP39 and *L. plantarum* D9.30 (Figure 3). Overall, *L. plantarum* (2), *P. pentosaceus* (2), and *Lact. paracasei* (1), isolated from various sources, showed peptidase activity higher than the third quartile towards both Leu- and Pro-*p*-Na (Figure S1).

### 3.4. Total Concentration of Peptides

**Figure 4:**
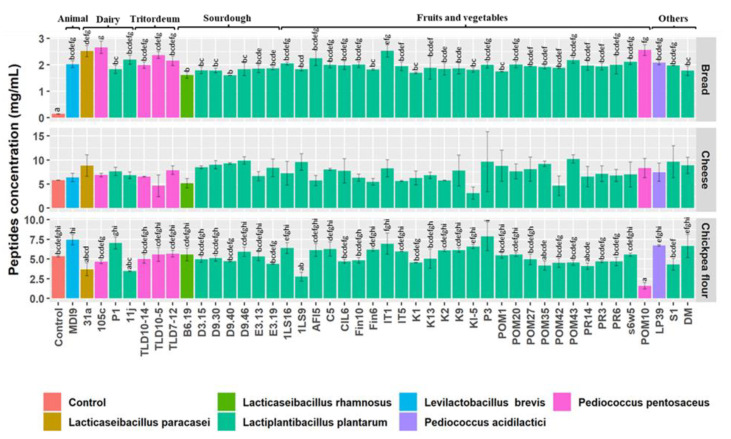
Peptide concentration values (mg/mL) in partially digested bread, cheese, and chickpea flour after 24 h incubation at 30 °C with 44 high-resistant lactic acid bacteria strains belonging to different species and isolated from different sources. Data are expressed as the mean of three separate analyses ± standard deviations. Bars with different superscript letters differ significantly (*p* < 0.05).

Aiming to investigate the digestive role of the 44 high-resistant strains, partially digested matrices were inoculated and incubated for 24 h at 30 °C (Figure 4). 

Due to low protein content, pomegranate and tomato matrices were excluded from this investigation. Regardless of strains and sources, the concentration of peptides was strongly dependent on the partially digested matrices. The initial peptides concentration in partially digested matrices were 0.14 ± 0.00 mg mL^−1^ (bread), 5.78 ± 0.01 mg mL^−1^ (cheese), and 5.38 ± 0.12 mg mL^−1^ (chickpea flour). When the bread was used as a substrate, all strains showed a substantial (*p* < 0.05) increase in peptide content which ranged from 1.60 ± 0.00 mg mL^−1^ (*L. plantarum* D9.40) to 2.66 ± 0.23 mg mL^−1^ (*P. pentosaceus* 105c). In cheese substrate, all the strains changed the concentration of the peptide but not significantly (*p* > 0.05). *L. plantarum* POM43 increased most of the peptides’ content in the partially digested cheese (10.20 ± 0.87 mg mL^−1^), whereas the maximum reduction was observed by *L. plantarum* KI-5 (3.12 ± 1.27 mg mL^−1^). After partially digesting chickpea flour, only *P. pentosaceus* POM10 was able to significantly (*p* < 0.05) reduce peptides’ content (1.60 ± 0.39 mg mL^−1^) compared to the control. Other strains demonstrated fluctuating values (2.77 ± 0.58 to 7.89 ± 1.81 mg mL^−1^) but were not significantly (*p* > 0.05) different from the control.

### 3.5. Total Free Phenolic Compounds

**Figure 5:**
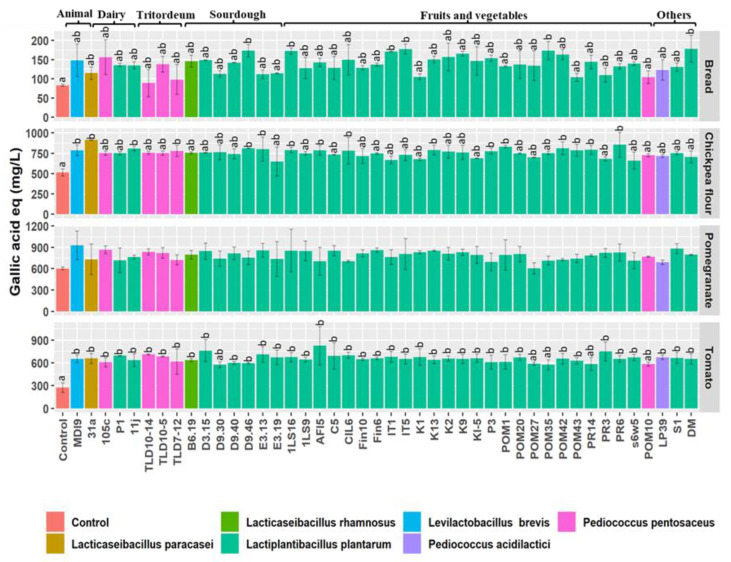
Total free phenolic compounds values (gallic acid equivalent mg/L) in partially digested bread, chickpea flour, pomegranate, and tomato after 24 h incubation at 30 °C with 44 high-resistant lactic acid bacteria strains belonging to different species and isolated from different sources. Data are expressed as the mean of three separate analyses ± standard deviations. Bars with different superscript letters differ significantly (*p* < 0.05).

The capacity of the 44 high-resistant strains to metabolize phenolic compounds in partially digested matrices was also evaluated (Figure 5). 

The concentration of total free phenolic compounds varied widely according to the partially digested matrices analyzed, with the highest initial values in pomegranate (602.8 ± 19.56 mg L^−1^), followed by chickpea flour (514.7 ± 45.19 mg L^−1^), tomato (273.4 ± 64.09 mg L^−1^), and bread (83.1 ± 2.46 mg L^−1^). In partially digested bread, six strains belonging to different species and sources markedly (*p* < 0.05) increased the total phenolic concentration. The highest values were recorded in partially digested bread incubated with *L. plantarum* DM (177.9 ± 35.11 mg L^−1^), whereas TLD10-14 had the lowest values (88.8 ± 35.97 mg L^−1^). A higher number of strains (17 strains) showed a significant increase in free phenolic compounds concentration in partially digested chickpea flour ranging from 649.2 ± 177.61 mg L^−1^ by *L. plantarum* P3 to 914.5 ± 1.53 mg L^−1^ by *Lact. paracasei* 31a. When compared to the control, other strains increased but not significantly (*p* > 0.05) the total free phenolic compounds concentration in partially digested chickpea flour. When pomegranate was used as a substrate, none of the strains showed significant capacity in releasing free phenolic compounds. Nevertheless, the highest total phenolic concentration was obtained after 24 h incubation by *Lev. brevis* MDI9 (928.4 ± 202.20 mg L^−1^) while the lowest was by *L. plantarum* POM27 (604.1 ± 78.65 mg L^−1^). On the contrary, an opposite trend was observed in partially digested tomato, in which most strains caused a relatively high significant (*p* < 0.05) increase in total free phenolic concentrations, except for six strains (*p* > 0.05). The highest total free phenolic concentrations were achieved by *L. plantarum* AF15 (827.4 ± 252.84 mg L^−1^).

### 3.6. Final Selection of the Most Promising Probiotic Candidates

Aiming to evaluate the best-performing strains based on multiple assays (resistance to GIT conditions, raffinose hydrolysis, peptidase activity, the total concentration of peptides and free phenolic compounds after 24 h incubation with partially digested matrices), a scoring approach was established. The data of all parameters were represented in boxplots to emphasize the strains present in the third quartile and assign them a positive score (score = 1) (Figure S1). Distinct species strains belonging to different sources were highlighted based on different assays and parameters (Table 1). 

The proportion of positive scores for a given assay was calculated by dividing the total cumulative score for an assay category divided by the number of parameters in the assay. One parameter (residual raffinose) was considered for raffinose hydrolysis. Only thirteen strains of *L. plantarum* obtained a maximum score (100%) for raffinose hydrolysis. The peptidase activity assay included two parameters (aminopeptidase activity PepN on Leu-*p*-Na and iminopeptidase activity PepI on Pro-*p*-Na). *L. plantarum* D9.46 (isolated from sourdough), *L. plantarum* K13 and *P. pentosaceus* POM10 (isolated from fruits and vegetables), *P. pentosaceus* 105c and *Lact. paracasei* 31a (isolated from milk) all showed the maximum peptidase activity score (100%). The OPA assay, reflecting the concentration of the total peptides, was evaluated based on three partially digested matrices. The highest score (67%) was obtained from *L. plantarum* P3, *Lb. plantarum* IT1, and *L. plantarum* POM43 (all isolated from fruits and vegetables), *Lact. paracasei* 31a (isolated from milk), *P. acidilactici* LP39, and *L. plantarum* DM (both isolated from other sources of isolation). On the other hand, the total free phenolic compounds assay relied on four partially digested matrices. The highest score for the release of total phenolic compounds (75%) was attained by *L. plantarum* 1LS16 and E3.13, isolated respectively from fruits and vegetables and sourdough (Table 1). 

## 4. Discussion

Being a source of microorganisms, mainly LAB, capable of promoting the balance of the gut microbiota and causing health-promotion effects on the host, probiotics deserve to be continuously investigated, as well as because of their sources of isolation and multi-functional properties [24]. Probiotics mostly used in commercial formulations have a human or dairy origin [25]. Nutritional, religious, and philosophical aspects [25] have bolstered the search for alternative sources of novel probiotic candidates from plant matrices, mainly fermented foods [26], broadening the possibilities for the use of LAB isolates in the development of various probiotic foods. Screening from high-isolation sources, which exploits different genetic potentials of selected LAB that originated from the intrinsic microbiota of distinct source ecosystems and are controlled by endogenous factors (e.g., moisture, temperature, pH and bioactive compounds), might be considered an advantageous approach for selecting promising probiotics candidates due to the close link between human health and fermented foods [27].

Since high survival rates and adaptation to the harsh conditions found in the GIT are the primary criteria for a probiotic to exert health benefits [28], our findings showed that, of the initial 446 strains, 44 strains maintained a viable cell population above ca. 7 Log CFU mL^−1^ after an in vitro simulation of the GIT for 6 h, turning them into potential probiotic candidates. This result does not contradict the fact that lesser performing LAB could have a high potential when co-cultivated [29]. Nevertheless, only the 44 strains previously selected as high-resistant strains were screened for their functional features in improving the host’s digestibility, aiming at choosing the best-performing strains to be used as dietary supplements. At first, the capability of strains to hydrolyze raffinose was evaluated. Raffinose, together with fructose, sorbitol, and mannitol, are the main carbohydrates of cereals and consequently in bread formulations [30]. Raffinose family oligosaccharides (RFOs) in foods are considered anti-nutritional factors since they are not degraded in the upper GIT, limiting their digestibility [31]. Indeed, the upper GIT lacks *α*-galactosidase, an intracellular enzyme able to degrade raffinose to galactose and sucrose as the final product of hydrolysis. Because of the non-degradation of raffinose, gastrointestinal symptoms may manifest, including abdominal discomfort, flatulence, and diarrhea [32]. Based on our results, all strains were able to thrive and markedly reduced raffinose content in Raf-MRS, with no common pattern for strains belonging to the same species or for strains isolated from the same source. These results were following what was previously stated by Harlé et al. [33], which was that raffinose catabolism in synthetic media demonstrated species- and strain-specificity, highlighting *L. plantarum* as one of the most efficient species to degrade raffinose. The strain-specificity of raffinose catabolism corroborates the necessity to screen a large number of strains to uncover interesting new candidates. Additionally, raffinose hydrolysis can highly improve protein digestibility during food fermentation [34]. Other features of LAB, linked to proteolytic activity, reduced protein complexity and exhibited a positive effect on protein digestibility but also liberated peptides and amino acids with a wide range of bioactivities. Since the proteolytic activity of LAB is well known, the role of peptidases to improve the digestibility of protein based-food matrices has been largely investigated [35,36]. Peptidases are enzymes that catalyze the cleavage of amino acids from the amino terminus of proteins and peptides. General aminopeptidase and proline-iminopeptidase have been isolated and genetically characterized in *L. helveticus* and both enzymes appear to be well conserved among lactobacillus sp. [37]. Our findings revealed the existence of both enzymes, mainly aminopeptidase, in all investigated strains at different extents, confirming species- and strain-specificity and emphasizing additional functional properties.

Digestibility, measured as the proportion of food that enters the digestive tract and is subsequently absorbed by the body, has legitimately become a vital factor in human health that also affects the desire for highly digestible foods among consumers and industries [38]. Digestion and fermentation of dietary protein are crucial steps, both to supply the organism with essential and non-essential amino acids and for the release of medium- and short-size peptides [19]. The same is true for the phenolic compounds, which are supposed to be available to perform their biological role. The bioavailability of certain dietary components may vary depending on the digestibility of their food matrix, their stability against various biochemical factors, and the involvement of gut microbes in the transformation process of dietary components in the gastrointestinal tract [39,40]. Clinical studies and in vivo experiments are necessary for a comprehensive evaluation of the bioavailability of any dietary component, but they are complicated, technically challenging, expensive, and may raise ethical concerns [41]. Therefore, a partial in vitro digestion procedure mimicking oral and gastric digestion was applied to the most representative food matrices. Cheese, bread, tomato, pomegranate, and chickpea flour were analyzed for peptides and phenolic compounds metabolism after oral and gastric digestive phases and successive incubation with the selected strains. After both treatments (digestion and fermentation), the variation of peptide and phenolic compound concentrations was reliant not only on the type and source of strains but also on the kind of digested food. In partially digested bread, all strains promoted peptides release, whereas in cheese and chickpea flour, amino acids release should be considered due to the reduction of peptide content. Proteolytic metabolism may involve many metabolic pathways. According to the literature, LAB have evolved complicated enzyme systems that result in the creation of small peptides or the release of free amino acids from big peptides in their immediate surroundings [42], which is confirmed in our findings. It is worth noting that high levels of undigested protein may result in an increase of pathogenic microorganisms with an associated higher risk of metabolic diseases [40]. This might underline the role of gut microbes or our potential probiotic candidates in hydrolyzing the proteins that escape host enzymatic digestion via extracellular proteases and peptidases, resulting in free amino acids and bioactive peptides [43]. Regarding phenolic content, all studied strains were able to enhance the release of bio-accessible free phenolic compounds in all partially digested matrices but to different extents. Polyphenols have recently gained a lot of interest in food due to their high antimicrobial and antioxidant features [44,45]. Phenolic compounds occur in foods mainly as esters, glycosides, and polymers [46]. Enzymes such as esterase, β-glucosidase, decarboxylase, and reductase harbored by LAB at differing levels can metabolize these compounds and increase their bio-accessibility at the gastrointestinal level to various extents, confirming our results and emphasizing another functional property for our selected strains [47].

The selection of a suitable probiotic product for patients has been a challenge because not all probiotics are equally beneficial or cost-efficient for different disorders. Multivariate statistical analysis has been deemed an effective method for identifying distinct characteristics of interest among a large number of bacterial isolates [25]. In our study, we pioneered a scoring approach that proved to be a useful tool to evaluate quantitively the probiotic potential of each candidate. Other advantages of this strategy might include reduced data set complexity and the possibility of evaluating a large number of LAB isolates for their potential to be used as probiotics. Based on our scoring, several strains of LAB showed diverse technological and functional probiotic-related properties specific to improve food digestibility. The next challenge might be the number of strains that should be included in probiotic formulations. Based on the available literature, a big gap existed on whether single strains are more or less effective than multi-strain combinations. According to McFarland et al. [48], in most cases, multi-strains mixtures were not significantly more effective than single-strain probiotics, whilst other studies revealed the advantages of multi-strains mixtures that may include synergistic effects of different strains performing different tasks in the mixture [49,50]. Therefore, the higher survival rates and best-performing strains might be incorporated, singularly or as a pool, in different pharmaceutical preparations and dietary supplements to improve food digestibility.

## 5. Conclusions

Our results confirmed that foods are potential sources of LAB with promising aptitudes to be exploited for possible probiotic purposes. These LAB isolates showed in vitro technological characteristics that could direct their incorporation into pharmaceutical preparations and dietary supplements to improve food digestibility. The scoring procedure developed could be a useful approach to identify distinct characteristics of interest in a high number of bacterial isolates and segregate isolates with the most promising aptitudes. Nevertheless, the most promising LAB isolates should be included in further in vivo investigations using cell lines or animal models to advance the definition of their health-promoting effects and confirm their potential for application as probiotics.

## Figures and Tables

**Table 1:** Score evaluations (%) of 44 high-resistant lactic acid bacteria strains, belonging to different species and isolated from different sources, based on the results collected from the assays carried out in this study.

		Raffinose Hydrolysis	Peptidase Activity		Peptides			Total Free Phenolics				Final Score (%)			
Species	Strains	Residual Raffinose	Leu-p-Na	Pro-p-Na	Bread	Cheese	Chickpea Flour	Bread	Chickpea Flour	Pomegranate	Tomato	Residual Raffinose	Peptidase Activity	Peptides	Total Free Phenolics
*Lactiplantibacillus plantarum*	K2	1	0	0	0	0	0	0	1	0	0	100	0	0	25
	P3	1	0	0	0	1	1	1	0	0	0	100	0	67	25
	POM1	1	0	0	0	1	0	0	0	0	0	100	0	33	0
	D9.46	1	1	1	0	1	0	1	1	0	0	100	100	33	50
	K13	0	1	1	0	0	0	1	0	0	0	0	100	0	25
	K9	1	0	1	0	0	1	0	0	0	1	100	50	33	25
	D9.40	1	0	1	0	1	0	0	0	0	0	100	50	33	0
	Fin10	1	0	1	0	0	0	0	0	0	0	100	50	0	0
	P1	0	0	0	0	0	1	0	0	0	1	0	0	33	25
	K1	0	0	0	0	0	0	1	0	0	0	0	0	0	25
	Fin6	0	0	0	0	0	1	0	0	0	1	0	0	33	25
	D3.15	0	1	0	0	0	0	0	0	1	1	0	50	0	50
	PR3	1	0	1	0	0	0	0	0	0	0	100	50	0	0
	D9.30	1	0	1	0	1	0	0	0	0	0	100	50	33	0
	E3.19	0	1	0	0	0	0	0	0	0	0	0	50	0	0
	1LS16	0	0	0	0	0	1	1	1	1	0	0	0	33	75
	E3.13	0	0	0	0	0	0	0	1	1	1	0	0	0	75
	IT1	0	0	0	1	0	1	1	0	0	1	0	0	67	50
	ILS9	0	0	0	0	1	0	0	1	0	1	0	0	33	50
	CIL6	1	0	0	0	0	0	0	0	1	0	100	0	0	25
	S1	0	0	0	0	1	0	0	0	1	0	0	0	33	25
	POM20	0	0	0	0	0	0	0	0	0	0	0	0	0	0
	POM35	0	1	0	0	1	0	1	1	0	0	0	50	33	50
	11j	0	1	0	1	0	0	0	1	0	0	0	50	33	25
	S6w5	0	1	0	1	0	0	0	0	1	0	0	50	33	25
	AFI5	0	0	0	1	0	0	0	0	1	1	0	0	33	50
	IT5	0	0	0	0	0	0	0	1	1	0	0	0	0	50
	DM	1	0	0	0	1	1	1	0	0	0	100	0	67	25
	C5	0	0	0	0	0	1	0	1	0	0	0	0	33	25
	POM27	0	0	0	0	0	0	1	0	0	0	0	0	0	25
	KI-5	0	0	0	0	0	1	0	0	0	1	0	0	33	25
	PR14	0	0	0	0	0	0	0	1	0	0	0	0	0	25
	PR6	0	0	0	0	0	0	1	0	0	0	0	0	0	25
	POM42	0	0	0	0	0	0	0	0	0	0	0	0	0	0
	POM43	0	0	0	1	1	0	0	0	0	0	0	0	67	0
*Lacticaseibacillus paracasei*	31a	0	1	1	1	1	0	0	1	0	0	0	100	67	25
*Lacticaseibacillus rhamnosus*	B6.19	0	0	1	0	0	0	0	0	0	0	0	50	0	0
*Levilactobacillus brevis*	MDI9	0	0	0	0	0	1	0	0	1	0	0	0	33	25
*Pediococcus acidilactici*	LP39	0	1	0	1	0	1	0	0	0	0	0	50	67	0
*Pediococcus pentosaceus*	105c	0	1	1	1	0	0	1	0	1	0	0	100	33	50
	POM10	0	1	1	1	0	0	0	0	0	0	0	100	33	0
	TLD10-14	0	0	0	0	0	0	0	0	1	1	0	0	0	50
	TLD10-5	0	0	0	1	0	0	0	0	0	1	0	0	33	25
	TLD7-12	0	0	0	1	0	0	0	0	0	0	0	0	33	0

## Data Availability

The data presented in this study are available on request from the corresponding author.

## Supplementary Materials

The following supporting information can be downloaded at: https://www.mdpi.com/article/10.3390/nu15061306/s1, Figure S1: Strains distribution according to their functional properties; Table S1: Lactic acid bacteria showing high-, intermediate-, or no-resistance under simulated gastrointestinal conditions; Table S2: List of high resistant lactic acid bacteria strains with their source and sub-source of isolation; Table S3: List of lactic acid bacteria used in this study.

## References

[1] Ghoshal U.C., Shukla R., Ghoshal U., Gwee K.A., Ng S.C., Quigley E.M. (2012). The gut microbiota and irritable bowel syndrome: Friend or foe?. Int. J. Inflamm..

[2] Yadav A., Chandra H., Maurya V.K. (2017). Probiotics: Recent advances and future prospects. J. Plant Dev. Sci..

[3] Tegegne B.A., Kebede B. (2022). Probiotics, their prophylactic and therapeutic applications in human health development: A review of the literature. Heliyon.

[4] Kerry R.G., Patra J.K., Gouda S., Park Y., Shin H.S., Das G. (2018). Benefaction of probiotics for human health: A review. J. Food Drug Anal..

[5] Sikorski Z.E. (2006). Chemical and Functional Properties of Food Components.

[6] Codex Alimentarius Commission, Food and Agriculture Organization of the United Nations, World Health Organization (2006). Probiotics in Food: Health and Nutritional Properties and Guidelines for Evaluation.

[7] Jäger R., Purpura M., Farmer S., Cash H.A., Keller D. (2018). Probiotic Bacillus coagulans GBI-30, 6086 improves protein absorption and utilization. Probiotics Antimicrob. Proteins.

[8] Stephens R.W., Arhire L., Covasa M. (2018). Gut microbiota: From microorganisms to metabolic organ influencing obesity. Obesity.

[9] Da Ros A., Polo A., Rizzello C.G., Acin-Albiac M., Montemurro M., Di Cagno R., Gobbetti M. (2021). Feeding with sustainably sourdough bread has the potential to promote the healthy microbiota metabolism at the colon level. Microbiol. Spectr..

[10] Mackie A., Mulet-Cabero A.I., Torcello-Gómez A. (2020). Simulating human digestion: Developing our knowledge to create healthier and more sustainable foods. Food Funct..

[11] Fioramonti J., Theodorou V., Bueno L. (2003). Probiotics: What are they? What are their effects on gut physiology?. Best Pract. Res. Clin. Gastroenterol..

[12] Filannino P., Di Cagno R., Gobbetti M. (2018). Metabolic and functional paths of lactic acid bacteria in plant foods: Get out of the labyrinth. Curr. Opin. Biotechnol..

[13] Vernaza M.G., Dia V.P., De Mejia E.G., Chang Y.K. (2012). Antioxidant and antiinflammatory properties of germinated and hydrolysed Brazilian soybean flours. Food Chem..

[14] Sanjukta S., Rai A.K. (2016). Production of bioactive peptides during soybean fermentation and their potential health benefits. Trends Food Sci. Technol..

[15] Ayivi R.D., Gyawali R., Krastanov A., Aljaloud S.O., Worku M., Tahergorabi R., Silva R.C.D., Ibrahim S.A. (2020). Lactic acidbacteria: Food safety and human health applications. Dairy.

[16] Fernández M.F., Boris S., Barbes C. (2003). Probiotic properties of human lactobacilli strains to be used in the gastrointestinal tract. J. Appl. Microbiol..

[17] Zárate G., Chaia A.P., González S., Oliver G. (2000). Viability and β-galactosidase activity of dairy propionibacteria subjected to digestion by artificial gastric and intestinal fluids. J. Food Prot..

[18] De Angelis M., Siragusa S., Berloco M., Caputo L., Settanni L., Alfonsi G., Amerio M., Grandi A., Ragni A., Gobbetti M. (2006). Selection of potential probiotic lactobacilli from pig feces to be used as additives in pelleted feeding. Res. Microbiol..

[19] Rizzello C.G., Nionelli L., Coda R., Gobbetti M. (2012). Synthesis of the cancer preventive peptide lunasin by lactic acid bacteria during sourdough fermentation. Nutr. Cancer.

[20] Brodkorb A., Egger L., Alminger M., Alvito P., Assunção R., Ballance S., Bohn T., Bourlieu-Lacanal C., Boutrou R., Carrière F. (2019). INFOGEST static in vitro simulation of gastrointestinal food digestion. Nat. Protoc..

[21] Church F.C., Swaisgood H.E., Porter D.H., Catignani G.L. (1983). Spectrophotometric assay using o-phthaldialdehyde for determination of proteolysis in milk and isolated milk proteins. J. Dairy Sci..

[22] Shannon E., Conlon M., Hayes M. (2022). The Prebiotic Effect of Australian Seaweeds on Commensal Bacteria and Short Chain Fatty Acid Production in a Simulated Gut Model. Nutrients.

[23] Mangiafico S.S. (2016). Summary and Analysis of Extension Program Evaluation in R.

[24] Alameri F., Tarique M., Osaili T., Obaid R., Abdalla A., Masad R., Al-Sbiei A., Fernandez-Cabezudo M., Liu S.Q., Al-Ramadi B. (2022). Lactic acid bacteria isolated from fresh vegetable products: Potential probiotic and postbiotic characteristics including immunomodulatory effects. Microorganisms.

[25] Rodrigues NP A., Garcia E.F., de Souza E.L. (2021). Selection of lactic acid bacteria with promising probiotic aptitudes from fruit and ability to survive in different food matrices. Braz. J. Microbiol..

[26] Vitali B., Minervini G., Rizzello C.G., Spisni E., Maccaferri S., Brigidi P., Gobbetti M., Di Cagno R. (2012). Novel probiotic candidates for humans isolated from raw fruits and vegetables. Food Microbiol..

[27] Steensels J., Gallone B., Voordeckers K., Verstrepen K.J. (2019). Domestication of industrial microbes. Curr. Biol..

[28] Polo A., Cappello C., Carafa I., Da Ros A., Baccilieri F., Di Cagno R., Gobbetti M. (2022). A novel functional herbal tea containing probiotic Bacillus coagulans GanedenBC30: An in vitro study using the Simulator of the Human Intestinal Microbial Ecosystem (SHIME). J. Funct. Foods.

[29] Ghosh S., Chowdhury R., Bhattacharya P. (2016). Mixed consortia in bioprocesses: Role of microbial interactions. Appl. Microbiol. Biotechnol..

[30] Gobbetti M., De Angelis M., Di Cagno R., Calasso M., Archetti G., Rizzello C.G. (2019). Novel insights on the functional/nutritional features of the sourdough fermentation. Int. J. Food Microbiol..

[31] Arora K., Carafa I., Fava F., Tuohy K.M., Nikoloudaki O., Gobbetti M., Di Cagno R. (2022). Sourdough performances of the golden cereal Tritordeum: Dynamics of microbial ecology, biochemical and nutritional features. Int. J. Food Microbiol..

[32] Teixeira J.S., McNeill V., Gänzle M.G. (2012). Levansucrase and sucrose phoshorylase contribute to raffinose, stachyose, and verbascose metabolism by lactobacilli. Food Microbiol..

[33] Harlé O., Falentin H., Niay J., Valence F., Courselaud C., Chuat V., Maillard M.B., Guédon É., Deutsch S.M., Thierry A. (2020). Diversity of the metabolic profiles of a broad range of lactic acid bacteria in soy juice fermentation. Food Microbiol..

[34] Montemurro M., Pontonio E., Gobbetti M., Rizzello C.G. (2019). Investigation of the nutritional, functional and technological effects of the sourdough fermentation of sprouted flours. Int. J. Food Microbiol..

[35] Di Cagno R., De Angelis M., Lavermicocca P., De Vincenzi M., Giovannini C., Faccia M., Gobbetti M. (2002). Proteolysis by sourdough lactic acid bacteria: Effects on wheat flour protein fractions and gliadin peptides involved in human cereal intolerance. Appl. Environ. Microbiol..

[36] Corsetti A., Gobbetti M., Smacchi E. (1996). Antibacterial activity of sourdough lactic acid bacteria: Isolation of a bacteriocin-like inhibitory substance from Lactobacillus sanfranciscoC57. Food Microbiol..

[37] Jensen M.P., Ardö Y. (2010). Variation in aminopeptidase and aminotransferase activities of six cheese related Lactobacillus helveticus strains. Int. Dairy J..

[38] Costantini A., Da Ros A., Nikoloudaki O., Montemurro M., Di Cagno R., Genot B., Gobbetti M., Rizzello C.G. (2022). How cereal flours, starters, enzymes, and process parameters affect the in vitro digestibility of sourdough bread. Food Res. Int..

[39] Manach C., Scalbert A., Morand C., Rémésy C., Jiménez L. (2004). Polyphenols: Food sources and bioavailability. Am. J. Clin. Nutr..

[40] Zhao J., Zhang X., Liu H., Brown M.A., Qiao S. (2019). Dietary protein and gut microbiota composition and function. Curr. Protein Pept. Sci..

[41] McDougall G.J., Fyffe S., Dobson P., Stewart D. (2005). Anthocyanins from red wine–their stability under simulated gastrointestinal digestion. Phytochemistry.

[42] Macedo A.C., Vieira M., Poças R., Malcata F.X. (2000). Peptide hydrolase system of lactic acid bacteria isolated from Serra da Estrela cheese. Int. Dairy J..

[43] Diether N.E., Willing B.P. (2019). Microbial fermentation of dietary protein: An important factor in diet–microbe–host interaction. Microorganisms.

[44] Tlais A.Z., Da Ros A., Filannino P., Vincentini O., Gobbetti M., Di Cagno R. (2021). Biotechnological re-cycling of apple by-products: A reservoir model to produce a dietary supplement fortified with biogenic phenolic compounds. Food Chem..

[45] Tlais AZ A., Lemos Junior WJ F., Filannino P., Campanaro S., Gobbetti M., Di Cagno R. (2022). How microbiome composition correlates with biochemical changes during sauerkraut fermentation: A focus on neglected bacterial players and functionalities. Microbiol. Spectr..

[46] Wojtunik-Kulesza K., Oniszczuk A., Oniszczuk T., Combrzyński M., Nowakowska D., Matwijczuk A. (2020). Influence of in vitro digestion on composition, bioaccessibility and antioxidant activity of food polyphenols—A non-systematic review. Nutrients.

[47] Tlais AZ A., Fiorino G.M., Polo A., Filannino P., Di Cagno R. (2020). High-value compounds in fruit, vegetable and cereal byproducts: An overview of potential sustainable reuse and exploitation. Molecules.

[48] McFarland L.V. (2021). Efficacy of single-strain probiotics versus multi-strain mixtures: Systematic review of strain and disease specificity. Dig. Dis. Sci..

[49] Timmerman H.M., Koning C.J., Mulder L., Rombouts F.M., Beynen A.C. (2004). Monostrain, multistrain and multispecies probiotics—A comparison of functionality and efficacy. Int. J. Food Microbiol..

[50] Pringsulaka O., Rueangyotchanthana K., Suwannasai N., Watanapokasin R., Amnueysit P., Sunthornthummas S., Sukkhum S., Sarawaneeyaruk S., Rangsiruji A. (2015). In vitro screening of lactic acid bacteria for multi-strain probiotics. Livest. Sci..

