# Supplementary material for: Identification and Selection of Prospective Probiotics for Enhancing Gastrointestinal Digestion: Application in Pharmaceutical Preparations and Dietary Supplements
Source: Nutrients. 2023 Mar 7;15(6):1306. doi: 10.3390/nu15061306 (PMC10053534; doi:10.3390/nu15061306)

Figure S1. Forty-four high-resistant lactic acid bacteria species strains isolated from different sources, distributed according to the residual raffinose concentration (g/L) after 24 h of incubation at 30 ºC in Raf-MRS (MRS broth medium supplemented with 20 g/L of raffinose) (A); distributed according to their peptidase activity (U) towards leucine- and proline *p*-nitroanilides (B); according to the peptides concentration values (mg/mL) after 24 h of incubation at 30 °C with partially digested bread, cheese and chickpea flour (C); and according to the total phenolic compounds values (gallic acid equivalent mg/L) after 24 h of incubation at 30 °C with partially digested bread, chickpea flour, pomegranate, and tomato (D). The upper and lower error bars in all the panels represent the lowest and highest values. The upper and lower boxes indicate the third and second quartiles, whereas the middle line shows the median of the data set.

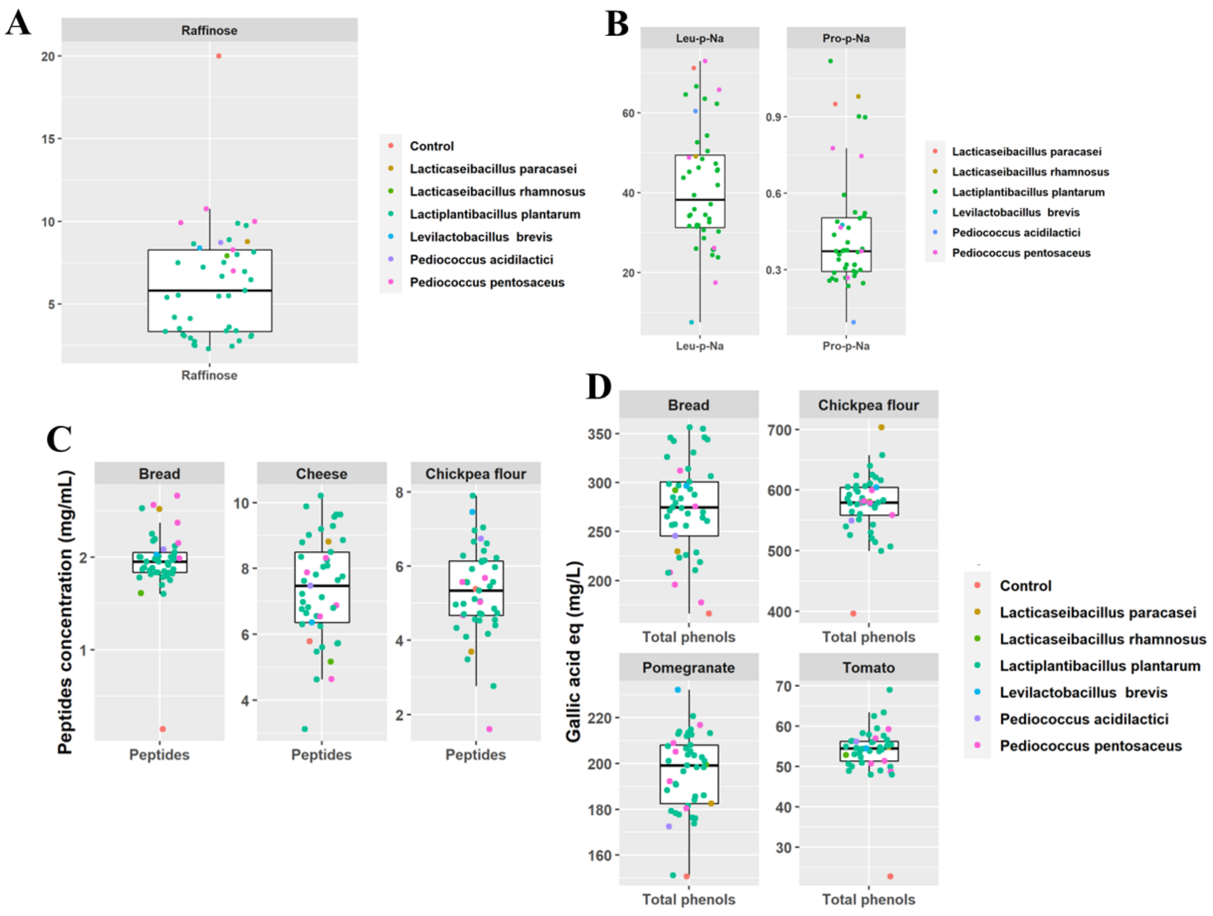

Supplement: Supplementary file 1 [file nutrients-15-01306-s001.zip › Figure S1.pdf]
