# Supplementary material for: Identification and Selection of Prospective Probiotics for Enhancing Gastrointestinal Digestion: Application in Pharmaceutical Preparations and Dietary Supplements
Source: Nutrients. 2023 Mar 7;15(6):1306. doi: 10.3390/nu15061306 (PMC10053534; doi:10.3390/nu15061306)
Supplement: Supplementary file 1 [file nutrients-15-01306-s001.zip › Table S1.pdf]

Table S1. Number of lactic acid bacteria strains that showed high-, intermediate- or no resistance under simulated gastrointestinal conditions. Data are obtained by comparing the cell density of each strain at the beginning of the incubation (t0) and after 3 and 6 h incubation with simulated gastric and intestinal fluids. (Cell density reduction (CDR) < 2 Log CFU/mL corresponds to high-resistant strains, 2 Log < CDR < 3 Log CFU/mL corresponds to intermediate-resistant strains and CDR > 3 Log CFU/mL corresponds to non-resistant strains.

Species	Total	High resistant	Intermediate resistant	Non-resistant
Levilactobacillus brevis	7	1	2	4
Lactobacillus curvatus	20	0	1	19
Limosilactobacillus fermentum	5	0	2	3
Lactobacillus gasseri	1	0	0	1
Lactobacillus helveticus	2	0	0	2
Lactobacillus parabuchneri	3	0	0	3
Lacticaseibacillus paracasei	34	1	1	32
Lactobacillus pentosus	13	0	3	10
Lactiplantibacillus plantarum	283	35	8	240
Lacticaseibacillus rhamnosus	15	1	1	13
Leuconostoc citreum	36	0	0	36
Leuconostoc mesenteroides	5	0	0	5
Leuconostoc pseudomesenteroides	7	0	0	7
Pediococcus acidilactici	1	1	0	0
Pediococcus parvulus	9	0	0	9
Pediococcus pentosaceus	5	5	0	0
**Grand Total**	**446**	**44**	**18**	**384**
