# Supplementary material for: Identification and Selection of Prospective Probiotics for Enhancing Gastrointestinal Digestion: Application in Pharmaceutical Preparations and Dietary Supplements
Source: Nutrients. 2023 Mar 7;15(6):1306. doi: 10.3390/nu15061306 (PMC10053534; doi:10.3390/nu15061306)
Supplement: Supplementary file 1 [file nutrients-15-01306-s001.zip › Table S2.pdf]

Table S2. List of high resistant lactic acid bacteria strains with their source and sub-source of isolation.

Number of strains	Species	Source and sub-source of isolation
1	*Lacticaseibacillus paracasei*	
	31a	Dairy (Milk)
1	*Lacticaseibacillus rhamnosus*	
	B6.19	Sourdough
35	*Lactiplantibacillus plantarum*	
	P1	Dairy (Cheese)
	11j	Dairy (Milk)
	C5	Fruits and vegetables (Carrot)
	CIL6	Fruits and vegetables (Cherry)
	Fin10, Fin6	Fruits and vegetables (Fennel)
	IT1, IT5	Fruits and vegetables (Grape)
	K1, K13, K2, K9, KI-5	Fruits and vegetables (Kiwi)
	P3	Fruits and vegetables (Papaya)
	1LS16, ILS9	Fruits and vegetables (Pineapple)
	PR14, PR3, PR6	Fruits and vegetables (Prune)
	AFI5, S6w5	Fruits and vegetables (Sauerkraut)
	POM1, POM20, POM27, POM35, POM40, POM42, POM43	Fruits and vegetables (Tomato)
	DM, S1	Other
	E3.13, E3.19, D9.30, D9.40, D9.46, D3.15	Sourdough
1	*Pediococcus acidilactici*	
	LP39	Other
5	*Pediococcus pentosaceus*	

	105c	Dairy (Milk)
	TLD10-14, TLD10-5, TLD7-12	Tritordeum
	POM10	Fruits and Vegetables (Tomato)
1	*Levilactobacillus brevis*	
	MDI9	Animal (*Drosophila melanogaster* intestine)
