# Supplementary material for: Identification and Selection of Prospective Probiotics for Enhancing Gastrointestinal Digestion: Application in Pharmaceutical Preparations and Dietary Supplements
Source: Nutrients. 2023 Mar 7;15(6):1306. doi: 10.3390/nu15061306 (PMC10053534; doi:10.3390/nu15061306)
Supplement: Supplementary file 1 [file nutrients-15-01306-s001.zip › Table S3.pdf]

Table S3. List of lactic acid bacteria used in this study.

Number of strains	Species	Source and sub-source of isolation
34	*Lacticaseibacillus paracasei*	
	31a, 25h, dII1, BBII10, bI5, HHI10, iiII9, iiII4, JJI8, kI12, MMII7, zzI6, eI3, ZZII6, wI10, 45j, 52i, 76d, 83e, 84f, LII1, 93j, 99a, 25g, WWI9, zzI4, zzI10	Dairy (Milk)
	FM14, FM4, FS12	Fruits and vegetables (Apple)
	S4d8	Fruits and vegetables (Sauerkraut)
	A2.14, A1.10, A3.23	Sourdough
15	*Lacticaseibacillus rhamnosus*	
	B6.19, B4.2, A4.14, A4.11, B3.69, B3.70, B3.39, B3.14, C6.4, C4.3, C4.8, D2.16, D2.19, D2.33, D10.3	Sourdough
283	*Lactiplantibacillus plantarum*	
	BEE1ST	Animal (*Apis milifera* intestine)
	DE12	Animal (*Drosophila melanogaster* intestine)
	FeO3	Animal (Feces)
	CB5, P1	Dairy (Cheese)
	11j	Dairy (Milk)
	F4-7	Flour (Oat)
	AVEF17	Fruits and vegetables (Avocado)
	C2, C5	Fruits and vegetables (Carrot)
	CIL6	Fruits and vegetables (Cherry)
	Fin10, Fin6	Fruits and vegetables (Fennel)
	IT1, IT5	Fruits and vegetables (Grape)
	K1, K13, K2, K9, KI-5	Fruits and vegetables (Kiwi)
	OE1	Fruits and vegetables (Olives)

	P3	Fruits and vegetables (Papaya)
	1LS16, ILS9, PJ16	Fruits and vegetables (Pineapple)
	PR14, PR3, PR6	Fruits and vegetables (Prune)
	AFI5, S6w5	Fruits and vegetables (Sauerkraut)
	POM1, POM20, POM27, POM35, POM40, POM42, POM43	Fruits and vegetables (Tomato)
	DM, S1	Other
	B4.3, B4.4, B6.18, A1.2, A2.59, A3.17, A3.40, A3.43, A3.46, A3.61, A3.62, A3.63, A3.68, A3.79, A4.13, A4.15, A4.18, A4.3, A4.5, A4.6, A6.1, A6.2, A6.3, E1.7, E2.22, E2.29, E2.39, E2.43, E2.45, E2.46, E2.50, E3.1, E3.13, E3.15, E3.17, E3.19, E3.21, E3.22, E3.53, E3.54, E3.55, E3.8, E4.16, E4.20, E4.9, E4.b, E6.16, C1.16, C2.15, C2.16, C2.24, C2.25, C2.26, C2.27, C2.28, C2.30, C2.32, C2.33, C2.34, C2.45, C2.46, C2.47, C2.48, C2.50, C2.51, C2.55, C2.71, C2.84, C3.11, C3.28, C3.33, C3.34, C3.38, C3.39, C3.40, C3.44, C3.46, C3.48, C3.51, C3.52, C3.53, C3.57, C3.61, C3.64, C3.66, C4.1, C4.10, C4.15, C4.18, C4.7, C5.9, C5.10, C5.3, C5.6, C6.10, C6.18, C6.6, C6.7, C6.9, D1.1, D1.10, D1.11, D1.12, D1.15, D1.16, D1.17, D1.18, D1.19, D1.2, D1.20, D1.3, D1.4, D1.9, D10.12, D10.13, D10.14, D10.15, D10.4, D10.5, D10.6, D10.7, D10.8, D2.11, D2.12, D2.13, D2.14, D2.17, D2.18, D2.20, D2.25, D2.26, D2.27, D2.28, D2.30, D2.34, D2.35, D2.36, D2.37, D2.38, D2.4, D2.40, D2.41, D2.42, D2.43, D2.44, D2.45, D2.46, D2.49, D2.5, D2.50, D2.6, D3.1, D3.10, D3.13, D3.14, D3.15, D3.17, D3.18, D3.19, D3.2, D3.20, D3.21, D3.22, D2.23, D3.24, D3.25, D3.26, D3.28, D3.29, D3.3, D3.30, D3.31, D3.32, D3.34, D3.35, D3.36, D3.37, D3.38, D3.39, D3.41,	Sourdough

	D3.42, D3.45, D3.46, D3.5, D3.6, D3.7, D3.8, D3.9, D4.1, D4,10, D4.11, D4.12, D4.13, D4.14, D4.15, D4.2, D4.3, D4.4, D4.6, D4.7, D4.8, D4.9, D4.e, D6.5, D6.7, D6.8, D9.1, D9.13, D9.14, D9.15, D9.16, D9.17, D9.18, D9.19, D9.2, D9.20, D9.21, D9.22, D9.23, D9.24, D9.25, D9.26, D9.28, D9.29, D9.30, D9.31, D9.32, D9.35, D9.36, D9.37, D9.38, D9.39, D9.40, D9.41, D9.42, D9.44, D9.45, D9.46, D9.5, D9.6, D9.7, D9.8, D9.9, TLD9-17	
20	*Lactobacillus curvatus*	
	VVI4, qqI5, qqII4, qqII6, qqII10, kI1, qqII5	Dairy (Milk)
	F5-10	Flour (Spelt)
	E6.20, E2.31, E2.41, E2.53, E2.61, E2.69, E2.70, E3.14, E3.24, E3.39, TLD7-11, TLD7-8	Sourdough
1	*Lactobacillus gasseri*	
	AI5	Dairy (Milk)
2	*Lactobacillus helveticus*	
	AII1, AII3	Dairy (Milk)
3	*Lactobacillus parabuchneri*	
	sI5, ZII9, bII5	Dairy (Milk)
13	*Lactobacillus pentosus*	
	O253, O3-S-8	Fruits and vegetables (Olives)
	D2.15, E1.4, E3.10, D2.1, D2.3, D2.10, E3.30, E3.34, E3.40, E6.17, E6.18	Sourdough
36	*Leuconostoc citreum*	
	E2.27, E2.28, E2.36, E2.37, E2.51, E2.59, E2.62, E3.31, E3.37, E3.41, E3.45, E3.48, E3.5, E3.68, E4.2, E4.6, E4.7, E4.8, E4.10, E4.12, E4.13, E4.15, E3.49, E10.1, E10.4, E10.5, E10.6, E10.3, E10.8, E10.9, E10.10, E10.11, E10.12, E10.13, E10.15, E10.18	Sourdough

5	*Leuconostoc mesenteroides*	
	E2.55	Sourdough
	S2d10, S2d5, S3d1, S3d7	Fruits and vegetables (Sauerkraut)
7	*Leuconostoc pseudomesenteroides*	
	E2.8, E2.9, E2.58, E2.24, E3.28, E3.33, E3.35	Sourdough
7	*Levilactobacillus brevis*	
	DIV18, MDI9	Animal (*Drosophila melanogaster* intestine)
	9e, 9f, bII7, 23f	Dairy (Milk)
	DIV7	Fruits and vegetables (Apple)
5	*Limosilactobacillus fermentum*	
	E3.44, E3.57, E2.14, E2.25, E2.52	Sourdough
1	*Pediococcus acidilactici*	
	LP39	Other
9	*Pediococcus parvulus*	
	S2w2, S2w6, S2w9, S2w1, S2w8, S4w10, S4w4, S5w1, S5w4	Fruits and vegetables (Sauerkraut)
5	*Pediococcus pentosaceus*	
	105c	Dairy (Milk)
	TLD10-10, TLD10-5, TLD7-12	Tritordeum
	POM10	Fruits and vegetables (Tomato)
